# Thrombocytopenia following kidney transplantation: a frequent, underestimated and potentially severe complication

**DOI:** 10.3389/fimmu.2025.1519256

**Published:** 2025-03-03

**Authors:** Cédric Léonard, Benjamin Taton, Estibaliz Lazaro, Pierre Merville, Jean-François Viallard, Lionel Couzi, Etienne Rivière

**Affiliations:** ^1^ Department of Internal Medicine, Haut-Lévêque Hospital, CHU de Bordeaux, Pessac, France; ^2^ Department of Nephrology Transplantation Dialysis Apheresis, CHU Bordeaux, Bordeaux, France; ^3^ CNRS “Immunoconcept” 5164, Université de Bordeaux, Bordeaux, France; ^4^ INSERM U1034, Bordeaux University, Pessac, France

**Keywords:** post-transplantation thrombocytopenia, thrombocytopenia, kidney transplantation, immune thrombocytopenia, bleeding, graft rejection

## Abstract

**Introduction:**

Cytopenias occur frequently after kidney transplantation but posttransplantation thrombocytopenia (PTTCP) frequency has rarely been reported.

**Methods:**

This monocenter, retrospective study aimed to describe PTTCP frequency, causes, treatments and outcomes. PTTCP was defined as thrombocytopenia with ≥2 platelet counts <100×10^9^/L after first month posttransplantation.

**Results:**

Among 2118 kidney-transplant recipients between 2002 and 2018, 189 (8.9%) developed PTTCP. Their mean platelet-count nadir was 51×10^9^/L [range 4-96×10^9^]; nadir was <50×10^9^/L for 87 (46.0%) patients. Main identified PTTCP etiologies were drugs (24.3%), or infectious diseases (20.1%; cytomegalovirus causing 79.4% of them), or unknown for 26 (13.7%). Bleeding rate was high (32.7%), with 40 (64.5%) severe episodes. During follow-up, 103 (54.5%) patients suffered graft loss or died at a median of 5.41 years post-PTTCP episode. Multivariate analyses retained a severe bleeding episode as being significantly associated with antiplatelet or anticoagulation therapy and pancytopenia, and age, creatininemia, transplantation-to-PTTCP interval and severe bleeding as significant risk factors for death or graft loss.

**Conclusion:**

PTTCP is frequently associated with severe bleeding, which is a risk factor for graft loss and death. Those findings suggest that the risk/benefit ratio of antiplatelet or anticoagulation therapies should be systemically evaluated for PTTCP patients.

## Introduction

Renal transplantation has become the best treatment option for patients with end-stage renal disease. However, it is associated with frequent and sometimes severe adverse complications. Posttransplantation cytopenias are among the most frequent (1), especially anemia, which is associated with poorer graft function (1–4). Post-transplantation thrombocytopenia (PTTCP), either isolated or associated with another cytopenia, has been much less investigated.

Thrombocytopenia is defined as a platelet count <150×10^9^/L but the threshold of 100×10^9^/L is of greater clinical interest as no bleeding event usually happens above this level (5). Thrombocytopenia increases the risk of bleeding in transplanted patients, especially when platelet counts are low: <30×10^9^/L or even <50×10^9^/L when combined with concomitant anticoagulation or anti-platelet therapies that are common in kidney-transplant recipients (6, 7). Intriguingly, neither this risk nor its cause has ever been assessed in the context of PTTCP.

Indeed, the results of only 2 studies suggested higher PTTCP rates of 20% (5, 8) to 66% (9) during year-1 posttransplantation. However, profound PTTCP, defined as platelet counts <50×10^9^/L, appears to be rare and has only been reported in 5% of patients after kidney transplantation (10, 11). Some PTTCP risk factors were identified in small studies: an episode of thrombocytopenia preceding transplantation, immunosuppressive maintenance therapy with everolimus, a supratherapeutic mycophenolic acid dose, deceased kidney donor, a previous graft-rejection episode, delayed graft function or infection (10, 12–17). Outcomes after PTTCP documentation have been poorly described. Some authors suggested enhanced graft failure induced by precautionary immunosuppression reduction, hypothesizing drug toxicity (18), but a higher frequency of acute cardiovascular events caused by stopping anti-platelet therapy or oral anticoagulation can be advanced, and even an increased risk of death, if a parallel is drawn with posttransplantation anemia.

This retrospective study was first designed to describe the PTTCP frequency among transplanted patients in southwestern France. Second, we analyzed the investigations to identify its underlying causes, bleeding-event rates and their severity, treatment(s) prescribed to increase platelet counts, and, finally, patients’ outcomes following a first PTTCP episode, notably the frequencies of rejection, graft failure and death during follow-up.

## Materials and methods

### Patient identification

All adult kidney-transplant recipients followed in our hospital’s Department of Renal Transplantation were included, using an electronic medical record (R@N software) that automatically registers medical follow-up and blood-test results (French Data-Protection Authority [CNIL], decision 2009−413, number 1357154; July 2, 2009). We screened every patient with ≥1 blood sample(s) with platelets <100×10^9^/L between August 2002 (first year of R@N-software use) and November 2018. Patients whose PTTCP occurred <1-month posttransplant were excluded because PTTCP probably reflected surgical complications or anti-thymocyte globulin initiation, as were those with only 1 low platelet count or incomplete medical files. We then collected data from eligible patients’ files starting after the first month posttransplantation. For patients with ≥2 PTTCP episodes, only details of the first episode were considered. A second PTTCP episode was defined as occurring during follow-up after complete resolution of a first episode (after >3 months with platelets >100×10^9^/L).

The following information about the PTTCP episodes was collected: platelet-count nadir, another cytopenia also documented at any time during the PTTCP episode (defined as hemoglobin <10 g/dL or neutropenia <1.8×10^9^/L), splenomegaly (splenic cranial-caudal height >14 cm on imaging), any bone-marrow aspirate and/or biopsy histology findings (cell density, number of megakaryocytes, dysplasia of any lineage or blasts and bone-marrow biopsy morphology, when available) and, if possible, the peripheral/central mechanism able to explain the thrombocytopenia. Anti-platelet antibody and ^111^indium-labeled platelet-scintigraphy results, only available for some patients, were also collected.

Anemia was defined as a hemoglobin level < 10 g/dL to highlight its significance given the various causes of anemia in renal transplant patients, particularly associated with impaired EPO secretion in chronic renal failure. The KDIGO guidelines recommend maintaining hemoglobin levels between 10 and 12 g/dL.

### Definition of patient groups according to their underlying PTTCP cause(s)

Patients were retrospectively assigned to 7 groups according to the underlying PTTCP etiology: 1) infection (concomitant pathogen presence), 2) drug induced (among drugs known to induce thrombocytopenia), 3) hypersplenism (splenomegaly >14 cm), 4) hematological disorder, 5) idiopathic thrombocytopenia purpura (ITP), 6) unknown cause, according to the main etiologies thought to induce thrombocytopenia, or 7) multiple causes, when ≥2 more were thought to independently trigger platelet decline simultaneously.

Among infectious causes, parvovirus B19 was incriminated only once, associating anemia with PTTCP, and only because the cytopenia resolved after intravenous immunoglobulin infusion (2). Sepsis-causing bacteria were retained when PTTCP resolved upon infection eradication. Cytomegalovirus (CMV) was deemed PTTCP-causative, when the infection was diagnosed according to international consensus guidelines (20), or PTTCP followed documented CMV infection with a normal bone-marrow smear during the first month of infection.

A therapeutic agent was held responsible when PTTCP resolved after withdrawal of a suspected drug. If several drugs were discontinued simultaneously and PTTCP resolved, all of them were considered PTTCP inducers.

Primary ITP was diagnosed according to international consensus guidelines (5). To simplify the analyses, CMV or other infectious agent-linked secondary ITP was considered to have only an “infection” etiology, not assigned to the ITP group.

We classified thrombotic microangiopathy-, hemophagocytic lymphohistiocytosis- or cirrhosis-inducing infections or drugs, as “hematologic disorder” or “hypersplenism” causes, not an infection or drug-induced PTTCP, including azathioprine-induced nodular regenerative hyperplasia (NRH).

For multiple-etiology or difficult-to-diagnose PTTCP episodes, a dedicated Adjudication Committee, comprised of nephrologists, immunologists and hematologists, determined the main cause(s), including all PTTCP episodes linked to ≥2 causes. Information on cardiovascular disease, graft rejection, renal failure and/or death were collected after PTTCP occurred; data collection was stopped after graft loss or for patients lost-to-follow-up.

### Bleeding score and thrombocytopenia severity

Kidney transplant patients received preventive anticoagulation with heparin until day 4 post-transplant, after which the treatment was discontinued. During the thrombocytopenia episode, the ITP bleeding score (**Supplementary File S1**) (19) was computed. It evaluates ITP-related bleeding severity, with a score >8 indicating hemorrhage and severe bleeding. Its items include the patient’s age, and various characteristics of cutaneous and mucosal (nose, throat, urinary, gynecologic, digestive or central nervous system) bleeding.

The need for packed red-cell transfusion(s) to counter bleeding or platelet transfusion(s), and any therapy given to raise the platelet count >100×10^9^/L were also recorded.

### Statistical analyses

Categorical variables are expressed as absolute counts (percentages) and continuous variables as medians (interquartile [IQR] ranges) or mean (range). For descriptive analyses, categorical variables were compared with χ^2^ or Fisher’s exact tests, as appropriate, and continuous variables with Student’s *t*-test. All statistical tests were 2-sided. Significance was defined as *P*<.05.

Graft and patient survivals post-PTTCP were estimated taking the beginning as PTTCP-episode onset, and the endpoint as the date of kidney failure, death or last known medical visit. The risk factors for severe bleeding were analyzed with Fine-Gray regressions, considering graft failure or patient death as competitive events, and a Cox proportional hazards model for those events.

Univariate analyses first selected risk factors for graft failure, bleeding and patient death as those achieving *P*<.2; they were included in multivariate models that were simplified by iterative backward elimination, retaining only the covariables with *P≤*.05.

## Results

### Characteristics of patients developing PTTCP

**Figure 1:**
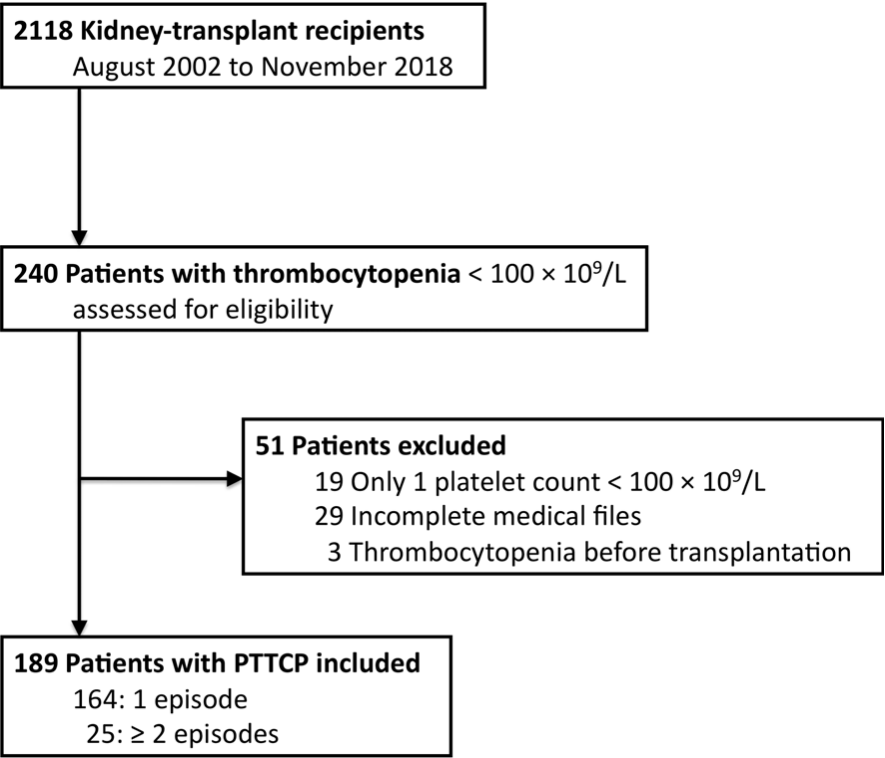
Flow chart of patient identification between August 2002 and November 2018. PTTCP, posttransplantation thrombocytopenia.

From August 2002 to November 2018, 2118 patients were followed in our hospital after kidney transplantation. Among them, 240 in our database were identified as having platelet counts <100×10^9^/L; 51 were excluded, as defined above, and 189 (8.9%) patients were finally retained for this analysis (**Figure 1**). Their characteristics at inclusion are detailed in **Table 1**: median [IQR] age at first PTTCP was 57 [23-81] years, 125 (66%) were men, and median transplantation-to-PTTCP interval was 17.5 [1-624] months.

**Table 1:** Inclusion characteristics of the 189 patients developing PTTCP.

Characteristic	Value
Age at first PTTCP, years	57 [23-81]
Men	125 (66%)
Platelet nadir	51×10^9^/L [4-96]
Transplantation-to-PTTCP interval, months	17.5 [1-624]
First kidney transplantation	152 (80.4)
Deceased-donor transplantation	169 (89.4)
Dialysis-to-transplantation interval, years	2.5 [0-24]
Time to transplant-function recovery, days	3 [1-60]
Initial nephropathies	
Unknown renal disease	32 (16.9)
Autosomal-dominant polycystic kidney disease	28 (14.8)
Diabetic, vascular or both nephropathy	25 (13.2)
Congenital	20 (10.6)
IgA nephropathy	19 (10.1)
Others^a^	65 (34.4)
Induction therapy	
Anti-thymocyte globulin	41 (21.7)
Anti-interleukin-2 receptor	102 (54)
Unknown	35 (18.5)
Others	8 (4.2)
Graft rejection before PTTCP^b^	49 (25.9)
T-cell-mediated rejection	17 (8.9)
Antibody-mediated rejection	24 (12.7)
Borderline rejection	8 (4.2)
On immunosuppressant(s) at PTTCP onset^c^	
CNI, MPA, steroids	84 (44.4)
CNI, MPA	28 (14.8)
CNI, mTORi, steroids	10 (5.3)
CNI, AZA, steroids	19 (10.1)
Any CNI-containing regimen	161 (85.2)
Any AZA-containing regimen	37 (19.6)
Any mTORi-containing regimen	25 (13.2)
Any MPA-containing regimen	124 (65.6)
Only 1 PTTCP episode	164 (86.8)
≥2 PTTCP episodes^d^	25 (13.2)

Results are expressed as n (%) or median [interquartile range (IQR)].

PTTCP, posttransplantation thrombocytopenia; CNI, calcineurin inhibitor; MPA: mycophenolic acid; mTORi, mammalian target of rapamycin inhibitor; AZA, azathioprine.

a Other causes were always <5: membranous glomerulonephritis, amyloidosis, atypical hemolytic uremic syndrome, urinary infection, genetic disease or drug toxicity.

b All rejections were biopsy-proven.

c Immunosuppressive therapy given on day-1 of PTTCP.

d A second PTTCP episode during follow-up was defined as occurring >3 months after the first episode had fully resolved, with platelets >100×10^9^/L for >3 months).

Among the 189 patients, 50 (26.5%) had isolated thrombocytopenia, 73 (38.6%) had PTTCP and anemia or neutropenia, and 66 (34.9%) had pancytopenia. Median platelet nadir was 51×10^9^/L [4-96]; nadirs were <10×10^9^/L for 9 (4.8%) patients, 11-50×10^9^/L for 78 (41.3%) and 51-100×10^9^/L for 102 (54%). Nadirs for 87 (46.0%) patients were <50×10^9^/L (i.e., 4.1% of all 2118 transplantees). Median PTTCP duration lasted 1 [2 days-204] months; the episode lasted <3 months for 129 (68.3%) patients and >12 months for 35 (18.5%). During follow-up, 151 (79.9%) PTTCP episodes resolved and 38 (20.1%) patients’ platelet counts never exceeded 100×10^9^/L or died before they could. Median follow-up from transplantation to death, graft failure or loss-to-follow-up was 7 [0-49] years. During that time, 25/189 (13.2%) patients had ≥2 PTTCP episodes.

### Main PTTCP etiologies

**Figure 2:**
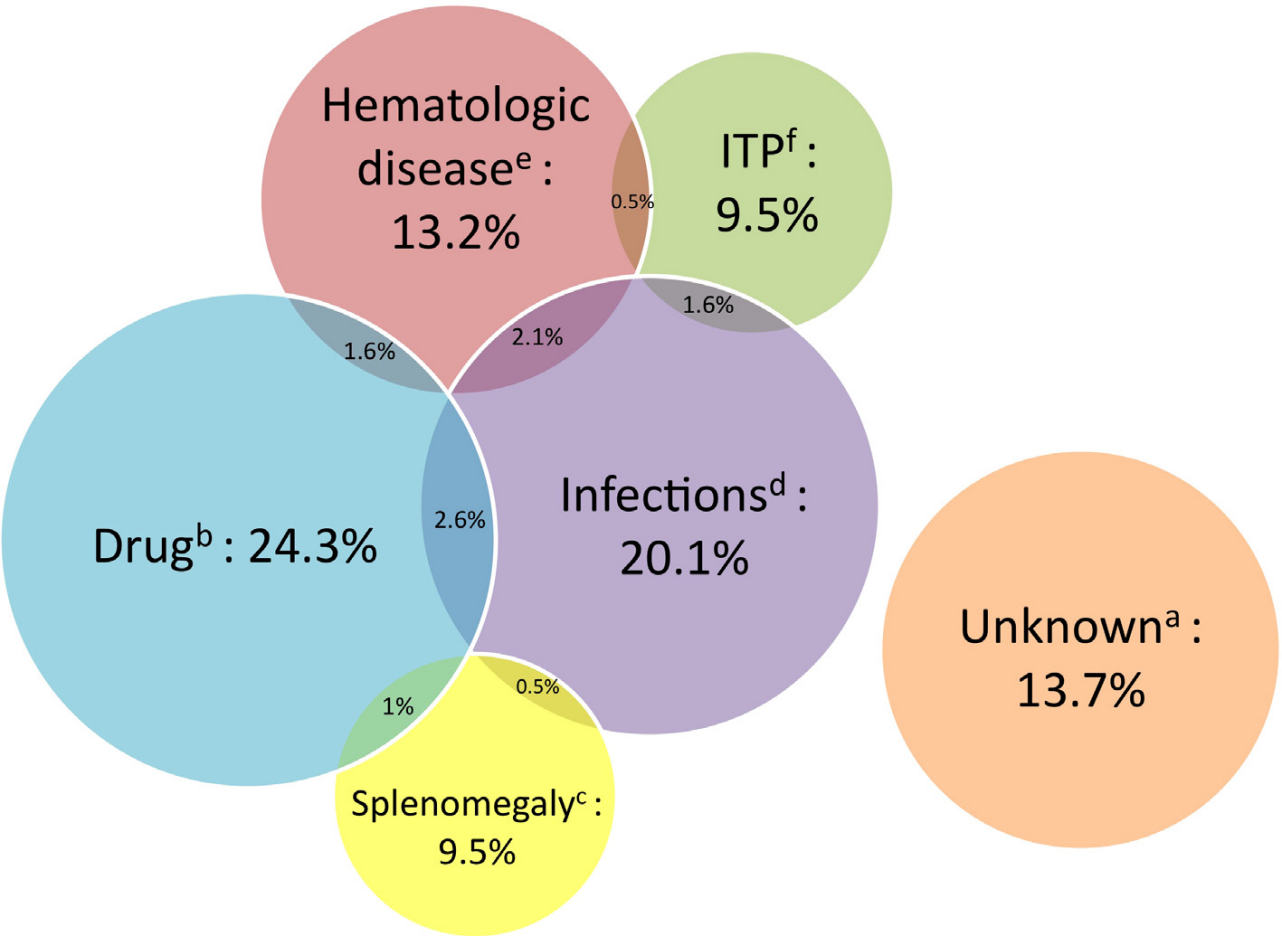
Main causes of thrombocytopenia following kidney transplantation (Venn diagram, *i.e.* circles are proportional to the number of patients in each group). ITP, idiopathic thrombocytopenia purpura; PTTCP, posttransplantation thrombocytopenia. ^a^Unknown origin (n=26). ^b^PTTCP induced by ≥1 drug(s) (n=46), defined as platelet-count normalization after stopping the suspected drug(s). ^c^Splenomegaly (n=18, 9.5%) defined as >14 cm on medical imagery. ^d^Infectious diseases (n= 38, 20.1%), for more details, see **Table 2**. ^e^Hematologic disease (n=25, 13.3%), for more details, see **Table 2**. ^f^ITP (n=18).

**Figure 2** summarizes the main identified causes of PTTCP. Notably, the Adjudication Committee identified ≥2 causes for 17 patients. The primary cause was drugs for 46, followed by infection for 38, unknown origin for 26, splenomegaly-related for 18 or hematologic disease for 25, and primary ITP for 18. One patient, not included in the figure, had only B9 and B12 deficiencies. Thirty-three (17.5%) patients experienced pretransplantation thrombocytopenia <100×10^9^/L and 13 (40%) of them relapsed with the same cause as previously.

CMV infection was incriminated in 34 (18%) patients: classed as the origin for 27 (79.4%) and 7 had multiple etiologies. Among the 34 kidney-transplant recipients with CMV infection, 26 had bone-marrow examinations during PTTCP; 21 (80.8%) of the latter had normal bone-marrow histology, suggesting increased platelet destruction. CMV-ITP often did not resolve after viral eradication; intravenous immunoglobulins or thrombopoietin-receptor agonists were successful for 7.

Azathioprine, prescribed to 37 PTTCP patients, was directly responsible for PTTCP in 16/37 (43.2%), notably for 8 with hepatic NRH. Other incriminated therapies were mycophenolate mofetil (MMF), valganciclovir, plasma exchange, intravenous immunoglobulins or linezolid (2 patients each) (**Table 2**).

**Table 2:** Thrombocytopenia etiologies for the 189 patients developing PTTCP.

Thrombocytopenia etiologies	Value n (%)
≥2 causes	17 (9)
Exclusively infection^a^	38 (20.1)
Cytomegalovirus	27 (14.3)
Sepsis	8 (4.2)
Other infections^b^	8 (4.2)
Only drug-related PTTCP	46 (24.3)
≥2 drugs suspected	10 (5.3)
Antiviral drugs (valaciclovir-valganciclovir)	6 (3.2)
Co-trimoxazole	5 (2.6)
Azathioprine^c^	3 (1.6)
Mycophenolate mofetil	9 (4.8)
Chemotherapy	6 (3.2)
Others	7 (3.7)
Only splenomegaly	18 (9.5)
Resulting from cirrhosis	7 (3.7)
Resulting from azathioprine-induced NRH	8 (4.2)
Others	3 (1.6)
Exclusively hematologic disorder	25 (13.2)
Thrombotic microangiopathy	11 (5.8)
Hemophagocytic lymphohistiocytosis	6 (3.2)
Myelodysplasia	5 (2.6)
Others	3 (1.6)
Exclusively primary ITP^d^	18 (9.5)
Including Evans syndrome	6 (3.2)

PTTCP, posttransplantation thrombocytopenia; NRH, nodular regenerative hyperplasia; ITP, idiopathic thrombocytopenia purpura.

a Including patients with 2 concomitant infections at PTTCP onset, e.g., cytomegalovirus and bacterial sepsis. Excluding infections causing cirrhosis, thrombotic microangiopathy, hemophagocytic lymphohistiocytosis or secondary ITP.

b Including tuberculosis, parvovirus B19 and human herpesvirus-8.

c Excluding the 8 patients with azathioprine-induced NRH.

d Excluding secondary infection or drug-induced ITP.

Among 13 thrombotic microangiopathies (including 2 with multiple etiologies), 10 were *de novo*: 4 were part of graft-rejection, 2 were calcineurin-inhibitor-induced, 1 had disseminated *Bartonella henselae* infection, 1 hemolytic uremic syndrome was linked to *Escherichia coli* and 2 others were atypical. Hemophagocytic lymphohistiocytosis was identified in 6 patients: 2 with posttransplantation lymphoproliferative disorders, 2 with sepsis, 1 with pulmonary tuberculosis, and 1 with disseminated *Bartonella* infection. Half of the 6 posttransplantation lymphoproliferative disorders were Epstein-Barr virus-related. In these patients, PTTCP was linked to bone marrow infiltration or chemotherapy, and one patient had splenomegaly.

PTTCPs appeared in 82 (43.4%) within year-1 posttransplantation. Among them, 25 (30.5%) were of infectious origin, including 22 (88%) CMV infections. After the first year, only 13/107 (12.1%) PTTCPs were infection-attributed and 6/107 (5.6%) CMV-related. Other causes did not differ between those 2 periods.

Specific interventions to raise the platelet count were started for 149/189 (78.8%) patients including immunosuppressive regimen dose-reduction for 97 (51.3%) patients, temporary/permanent withdrawal of a non-immunosuppressive drug for 51 (34.2%) and anti-infectious drug initiation for 49 (32.9%) (**Table 3**).

**Table 3:** Bleeding-complication characteristics of the 189 patients’ PTTCP episodes, their treatments and outcomes.

Characteristic	Value, n (%)^a^
Bleeding	62 (32.8)
Khellaf bleeding score, median [IQR]^b^	15 [1-30]
Severe (Khellaf bleeding score >8)	40 (21.2)
Digestive	15 (7.9)
Urogenital	10 (5.3)
Cerebral	5 (2.6)
Causing death	6 (3.2)
Need to stop anticoagulation or anti-platelet therapy^c^	38 (20.1)
Packed red-cell transfusion^d^	33 (17.5)
Platelet transfusion	46 (24.3)
Outcome after PTTCP	
Graft survival after PTTCP, years	5.41 [0-49]
Graft duration posttransplant, years	7 [0.1-49]
Graft rejection^e^	34 (18)
Cardiovascular event^f^	40 (21.2)
Graft loss	75 (39.7)
Any-cause death	28 (14.8)
Death or graft loss	103 (54.5)

Results are expressed as n (%) or median [interquartile range (IQR)].

PTTCP, posttransplantation thrombocytopenia.

a Unless stated otherwise.

b Calculated at any time during the PTTCP episode and only the highest score was retained.

c Temporarily or permanently.

d Only packed red-cell transfusions for bleeding were analyzed. Transfusions for anemia unrelated to bleeding were not considered.

e All rejections were biopsy-proven.

f Death from a cardiovascular cause: myocardial infarction, unstable angina, transient ischemic attack, stroke or need for coronary artery revascularization.

### Suspected thrombocytopenia mechanism

Anti-platelet antibodies were detected in 16/57 (28.1%) tested patients, suggesting an autoimmune mechanism for the thrombocytopenia, even though this test lacks specificity and sensitivity (5). A bone-marrow smear was obtained for 121 (64.0%) patients, among whom 14 also had bone-marrow biopsies. Eighty-seven (71.9%) of the 121 patients had normal findings; notably, medullar cell density was normal or increased in 97 of them (80.2%). Because the thrombocytopenia mechanism was still unclear, platelet scintigraphy was done for 9 patients; it revealed shortened platelet life-span caused by increased platelet destruction in only 1 patient. Finally, although we agree making some diagnoses was not clear cut, those observations led us to conclude that hematopoiesis was affected in 54 (28.6%) patients, platelet destruction was enhanced in 128 (67.7%) and a mixed mechanism was involved in 7 (3.7%).

### Bleeding complications

Sixty-two (32.8%) patients suffered bleeding complications when their platelet counts were <100×10^9^/L; bleeding was severe (defined as a bleeding score >8) in 40 (64.5%) of them. Six of (15%) those 40 patients died: 2 from hemorrhagic strokes, 3 from hemoptysis and 1 from digestive bleeding.

Patients experiencing ≥1 severe bleeding episode(s), compared to those without, respectively, had: lower platelet-count nadirs (40×10^9/L^ vs 53×10^9^/L; *P*=.005), higher percentages with ≥1 episode(s) of very low platelet counts <10×10^9^/L (5/40 [12.5%] vs 4/149 [2.7%]; *P*=.01), and requiring more packed red-cell transfusions (29/40 [72.5%] vs 4/149 [2.7%]; *P*=6×10^-24^) and more platelet infusions (24/40 [60%] vs 22/149 [14.8%]; *P*=1.1×10^-8^).

Patients who experienced at least one episode of thrombocytopenia, with a platelet count <50×10^9^/L, exhibited more severe bleeding compared to those with a platelet count >50×10^9^/L (29.9% versus 12.7%, p = 0.003). This trend was even more pronounced in patients with platelet counts < 10 × 10^9^/L, where the rates of severe bleeding were 12.5% compared to 2.7% (p = 0.01). Among those in the severe bleeding group, a greater proportion required cessation of anticoagulant or antiplatelet therapies compared to patients either not experiencing severe bleeding or with no bleeding incidents (20 out of 40 [50%] versus 18 out of 149 [12%], p < 0.00005). Univariate analyses (**Table 4**) selected the following covariables as being significantly associated with a severe bleeding episode during PTTCP: pancytopenia, taking azathioprine at PTTCP onset and concomitant antiplatelet or anticoagulation therapy at that time. According to our multivariate analysis, severe bleeding during PTTCP was significantly and independently associated with pancytopenia and antiplatelet or anticoagulation therapy at that time.

**Table 4:** Univariate and multivariate analyses of risk factors for severe bleeding^a^ during PTTCP. .

Covariable	Hazard ratio	95% CI	*P* ^b^
Univariate analysis			
Donor’s age (per year)	1.02	0.99-1.04	.16
Recipient’s age (per year) Dialysis duration (per year) Recipient’s sex	1.02 0.99 0.94	0.99-1.04 0.92-1.08 0.48-1.83	.16 .95 .86
HLA-sensitized (yes vs no)	1.01	0.53-1.90	.99
Expanded criteria donor (yes vs no)	1.43	0.75- 2.75	.27
*De novo* DSA (yes vs no)	0.48	0.15 -1.54	.22
Rejection before PTTCP (yes vs no)	1.50	0.77-2.89	.23
Transplantation-to-PTTCP interval (per year) Creatininemia at PTTCP onset (per mg/dL)	1.01 1.02	0.98-1.05 0.92-1.12	.53 .73
PTTCP etiology			
Infection (yes vs no)	1.01	0.52-1.98	.97
Toxic (yes vs no)	0.96	0.50-1.81	.90
Splenomegaly (yes vs no)	1.8	0.90-3.61	.098
Hematologic (yes vs no)	1.43	0.67-3.03	.35
Immunologic (yes vs no)	0.69	0.22-2.14	.52
Pancytopenia	1.95	1.05-3.27	**.036**
Cirrhosis	1.77	0.73-4.31	.21
Azathioprine use at PTTCP onset	2.55	1.23-5.32	**.01**
Antiplatelet or anticoagulation therapy	2.05	1.10-3.86	**.026**
Multivariate analysis^c^			
Pancytopenia	1.97	1.05-3.70	**.035**
On antiplatelet or anticoagulation therapy	2.2	1.15-4.19	**.017**

PTTCP, posttransplantation thrombocytopenia; HLA, anti-human leukocyte antigen; DSA, donor specific HLA antibodies.

a Defined as Khellaf bleeding score >8.

b Bold values indicate statistically significant associations, ie, *P*<.05.

c Tested covariables were covariates sufficiently associated with the outcome (*P*<.2) in univariate analysis.

### Post-PTTCP risk factors for graft loss or death

During follow-up, graft rejection occurred in 34 (18%) patients, graft loss in 75 (39.7%) and 28 (14.8%) died. Mostly after the PTTCP episode resolved, 103 (54.5%) patients met the composite outcome of graft loss or death, within a median of 5.4 [0-49] years (**Table 3**).

When comparing patients with subsequent graft loss or death to those without, the lowest recorded platelet counts were similar between the two groups (49.5×10^9^/L versus 52.1×10^9^/L; p = 0.55). Additionally, the proportion of patients with platelet counts <50×10^9^/L was comparable (48 out of 103 [46.6%] versus 37 out of 89 [41.6%]; p = 0.64). Univariate analyses (**Table 5**) identified the following risk factors as being significantly associated with composite graft loss or death: donor and recipient ages, previous rejection episode before PTTCP, transplantation-to-PTTCP interval, PTTCP occurring at least 9 years posttransplantation, creatininemia at PTTCP diagnosis, bleeding during PTTCP and severe bleeding. According to our multivariate analyses, the risk factors significantly and independently associated with graft loss or death were: recipient’s age, transplantation-to-PTTCP interval, creatininemia at PTTCP onset and severe bleeding during PTTCP. Thirty-three patients (17.4%) experienced graft rejection following the PTTCP episode. Among these, 22 patients (64.7%) had a dose reduction of immunosuppressive drugs, primarily MMF, or an interruption of azathioprine, which occurred significantly more frequently (p<0.05) than in patients without graft rejection (26.4%).

**Table 5:** Univariate and multivariate analyses of risk factors for graft and patient survivals for the 189 PTTCP patients.

Analysis Covariable	Hazard ratio	95% CI	*P* ^a^
Univariate			
Donor’s age (per year)	1.02	1.01-1.04	**.007**
Recipient’s age (per year)	1.02	1.01-1.04	**.007**
Recipient’s sex	1.16	0.76 -1.79	.49
Graft rank at PTTCP onset	1.13	0.55 -2.30	.74
HLA-sensitized (yes vs no)	0.96	0.64-1.44	.85
Expanded criteria donor (yes vs no)	1.32	0.88-1.97	.177
*De novo* DSA (yes vs no)	1.00	0.57-1.76	.99
Previous rejection before PTTCP (yes vs no)	1.58	1.03-2.43	**.03**
Transplantation-to-PTTCP interval (per year)	1.03	1.01-1.06	**.01**
Transplantation-to-PTTCP interval, year			
<3	Reference	—	—
3-6	1.69	0.95-3.00	.073
6-9	1.06	0.58-1.96	.84
>9	1.83	1.10-3.04	**.02**
PTTCP cause			
Infection (yes vs no)	0.90	0.60-1.38	.64
Toxic (yes vs no)	1.29	0.9-1.92	.20
Splenomegaly (yes vs no)	1.16	0.69-1.95	.57
Hematologic (yes vs no)	1.14	0.70-1.86	.59
Immunologic (yes vs no)	1.72	0.95-3.08	.07
Creatininemia at PTTCP onset (per mg/dL)	1.15	1.09-1.22	**1.5×10^-6^**
Bleeding (yes vs no)	1.86	1.22-2.85	**.004**
Severe bleeding^b^ (yes vs no)	2.53	1.59-4.04	**9.2×10^-5^**
Multivariate^c^			
Recipient’s age (per year)	1.02	1.01-1.04	**.013**
Transplantation-to-PTTCP interval (per year)	1.03	1.01-1.06	**.014**
Creatininemia at PTTCP onset (per mg/dL)	1.19	1.11-1.26	**4.3×10^-7^**
Severe bleeding^b^ (yes vs no)	2.22	1.38-3.56	**9.8×10^-4^**

PTTCP, posttransplantation thrombocytopenia; HLA, specific anti-human leukocyte antigen; DSA, donor HLA antibodies.

a Bold values indicate statistically significant associations, ie, *P*<.05.

b Defined as a Khellaf bleeding score >8.

c Tested covariables were covariates sufficiently associated with the outcome (p<0.2) in univariate analysis.

## Discussion

This monocenter study, based on 2118 kidney-transplant recipients from 2002 to 2018, focused on PTTCP, defined as platelet count <100×10^9^/L. Among them, 189 (8.9%) developed PTTCP, that appeared in 43% within year-1 posttransplant, representing 4.1% of all transplantees with platelet nadirs <50×10^9^/L. That rate was lower than previously reported (10–12), which ranged from 20% to 66%, mainly attributable to longer follow-up (median 7 years herein) and strict inclusion criteria (lower PTTCP-defining threshold and exclusion of PTTCP occurring during the first month posttransplantation). Infections (20.1%) and drug-induction (24.3%) were primary PTTCP etiologies, often concomitant, as previously reported (1, 3). Although ~80% of PTTCP episodes resolved, severe bleeding occurred in nearly one-third of the patients, associated with significant mortality or graft loss.

Importantly, systematic evaluation of primary and secondary PTTCP etiologies is essential, because 8.9% of patients experiencing PTTCP had 2 concurrent causes. The drugs most frequently associated with PTTCP were MMF and azathioprine. We believe that the discontinuation of any suspected drug should be systematically evaluated in terms of the benefit/risk balance. Notably, proton-pump inhibitors were never responsible for thrombocytopenia (22) and MMF dose-reduction improved platelet levels only for neutropenia-associated PTTCP. CMV infection was the leading infectious PTTCP etiology, representing ~80% of patients with infections, especially within year-1 posttransplant. For patients with thrombocytopenia episodes pretransplant, the PTTCP cause was the same for 40% of episodes. It is also important to note that we had to simplify the analysis of the numerous underlying causes. For instance, we did not classify thrombotic microangiopathy (TMA), hemophagocytic lymphohistiocytosis (HLH), or cirrhosis resulting from an underlying infection as “infection-induced PTTCP”.

Defective hematopoiesis (28.6%), increased platelet destruction (67.7%) and mixed mechanisms (3.7%) were other PTTCP etiologies. In our population, 9.5% of kidney transplantees had ITP, a rate higher than previously reported (23–28). Our experience highlights the challenge of diagnosing ITP in kidney-transplant recipients due to confounding factors and emphasized the importance of bone-marrow histology.

Despite bone marrow-based ITP diagnoses, scintigraphy often revealed normal platelet lifetimes, challenging the ITP diagnosis (9). For example, CMV-induced thrombocytopenia had distinct mechanisms. Pertinently, bone-marrow morphology was normal during PTTCP episodes in ~80% of the samples obtained from 24/34 of CMV-infected kidney-transplant recipients, 21 suggesting increased platelet destruction. PTTCP did not always resolve after virus eradication and some of them required intravenous immunoglobulins or thrombopoietin-receptor agonists to successfully treat their PTTCPs. It is important to note that the mechanism behind CMV-induced PTTCP is not fully understood (29–32) and that our study was not designed to address that question.

Bleeding complications occurred in about one-third of the patients and were severe in two-thirds of them, as defined by Khellaf bleeding scores >8 (median score 15), which is a well-recognized score (19). Those severe bleeding episodes were mostly gastrointestinal bleeding, with major blood loss or hemorrhagic shock, or brain hemorrhage; 6 of those patients died. Severe bleeding was associated with lower nadir platelet counts and high red-cell (for three-quarters) and platelet transfusion requirements (for almost two-thirds). Surprisingly, no association was found between PTTCP etiology and bleeding risk but the relatively low identification rate of severe PTTCP causes might introduce bias. Notably, our multivariate analyses retained severe bleeding as being significantly and independently associated with concurrent antiplatelet or anticoagulant therapies and ongoing pancytopenia. Given that prescription of those medications is a modifiable risk factor, it is very important to assess their benefit/risk in patients with TTP. When possible, their discontinuation could be a lever used to prevent severe bleeding. Of note, in our cohort, non-severe bleedings were not associated with the use of these medications. This is probably because non-severe bleedings were usually mild allowing the continuation of these drugs, whereas severe bleedings were more prevalent with overt mucosal bleeding that led to treatment discontinuation.

Over half of the patients experienced graft loss or died a median of 5.4 years after the first PTTCP episode. Multivariate analysis retained recipient’s age, transplantation-to-PTTCP-diagnosis interval, creatininemia at PTTCP diagnosis, and severe bleeding during the PTTCP episode as risk factors for those events. Severe bleeding was the direct cause of death for only 6 patients. Unfortunately, this study was not designed to determine the relationship between those events and the PTTCP episode itself. Hence, for that small subpopulation, severe bleeding might simply reflect greater individual fragility or underlying comorbidities. Regardless of the severe bleeding-graft loss link, patients experiencing PTTCP require close monitoring during the episode and over the following years. Iterative rejection episodes induced by immunosuppressant-dose reduction to address thrombocytopenia could contribute to graft losses. Indeed, 33 patients experienced graft rejection following PTTCP, and immunosuppressant, mainly MMF, dose-reduction for about two-thirds of them. Previously reported results suggested an association between MMF-dose reduction and acute rejection risk (18, 21), emphasizing the importance of maintaining dose reduction only when platelet counts rise. A revaluation of the immunosuppressive regimen is necessary when drug reduction or discontinuation is aimed at improving platelet numbers.

The study’s strengths include its comprehensive identification of patients who benefited from prolonged median follow-up of 7 years. However, the retrospective single-center design might limit the generalizability of findings. Also, the absence of a control group makes it challenging to precisely identify risk factors preceding PTTCP. Nevertheless, this analysis provided comprehensive insights into PTTCP outcomes, bleeding risks and long-term consequences.

## Conclusion

PTTCP is a frequent and serious complication in kidney-transplant recipients, often accompanied by bleeding complications. Our study revealed an underestimation of PTTCP during follow-up that affected 8.9% of our population. Infections, particularly CMV, and inducing drugs (MMF, azathioprine) were identified as the most common etiologies. Bleeding complications occurred in nearly one-third of the kidney transplantees, with two-thirds experiencing severe bleeding. Multivariate analysis retained ongoing antiplatelet or anticoagulation therapy and pancytopenia as significant risk factors for severe bleeding, and age, creatininemia and severe bleeding during PTTCP were identified as risk factors for graft loss or death, affecting 54.5% of our population. The study findings underscore the need for careful monitoring and evaluation of patients with PTTCP to mitigate adverse outcomes, including graft loss or death, even if the link between the two events is not established.

## Data Availability

The raw data supporting the conclusions of this article will be made available by the authors, upon reasonnable request.

## Supplementary material

Supplementary Table 1 Bleeding score adapted from Khellaf et al. (19). **^a^** For these items, only the highest value was taken into account. Severe bleeding is defined as a bleeding with a score >8.
