# Supplementary material for: Thrombocytopenia following kidney transplantation: a frequent, underestimated and potentially severe complication
Source: Front Immunol. 2025 Mar 3;16:1519256. doi: 10.3389/fimmu.2025.1519256 (PMC11911467; doi:10.3389/fimmu.2025.1519256)
Supplement: Supplementary Table 1 — Bleeding score adapted from Khellaf et al. (19). a For these items, only the highest value was taken into account. Severe bleeding is defined as a bleeding with a score >8. [file Table1.docx]

**SUPPLEMENTAL DATA**

**Supplementary Table 1.** Bleeding score adapted from Khellaf et al.^19^

| Item | Points |
| --- | --- |
| Age^a^ |  |
| >65 years | 2 |
| >75 years | 5 |
| Cutaneous bleeding^a^ |  |
| Localized petechial purpura (legs) | 1 |
| Localized ecchymotic purpura | 2 |
| 2 petechial purpura locations | 2 |
| Generalized petechial purpura | 3 |
| Generalized ecchymotic purpura | 4 |
| Mucosal bleeding |  |
| Unilateral epistaxis | 2 |
| Bilateral epistaxis | 3 |
| Hemorrhagic oral bullae, spontaneous gingival bleeding or both | 5 |
| Gastrointestinal bleeding^a^ |  |
| Gastrointestinal bleeding without anemia | 4 |
| Gastrointestinal bleeding with acute anemia (>2 g Hb decrease in 24 h) an/or shock | 15 |
| Urinary bleeding^a^ |  |
| Macroscopic hematuria without anemia | 4 |
| Macroscopic hematuria with acute anemia (>2 g Hb decrease in 24 h) and/or shock | 10 |
| Genitourinary tract bleeding^a^ |  |
| Major meno-/metrorrhagia without anemia^a^ | 4 |
| Major meno-/metrorrhagia with acute anemia (>2 g Hb decrease in 24 h) and/or shock | 10 |
| Central nervous system bleeding or other life-threatening hemorrhage | 15 |

***^a^*** *For these items, only the highest value was taken into account.*

*Severe bleeding is defined as a bleeding with a score >8.*
